# Lost in another language: a case report

**DOI:** 10.1186/s13256-021-03236-z

**Published:** 2022-01-22

**Authors:** Husam K. Z. Salamah, Eva Mortier, Renske Wassenberg, Jacqueline J. M. H. Strik

**Affiliations:** 1 Department of Psychiatry and Department of Psychology, Maastricht University Medical Center+, P.O. Box 5800, 6202 AZ Maastricht, the Netherlands; 2 School for Mental Health and Neuroscience (MHeNs), University of Maastricht, Maastricht, the Netherlands

**Keywords:** Language switch, Foreign language syndrome, Anesthesia, Emergence delirium, Postoperative, Non-native, Case report

## Abstract

**Background:**

In foreign language syndrome, patients switch from their native language and fixate for a period of time on a second language. There have been few reported cases. The language switch typically occurs postoperatively and spontaneously resolves after a short period of time. The primary cause of this switching remains unclear. There is speculation about the involvement of anesthesia, but its specific influence remains unclear.

**Case presentation:**

A 17-year-old Dutch Caucasian male lost the ability to understand and speak Dutch for 24 hours after an orthopedic surgery, combined with a brief confused state including disorientation of place and the inability to recognize his parents. During the period, he communicated in English, which he had learned during school classes but had never spoken outside of school. Further follow-up, including neuropsychological examination, revealed no indication of cognitive impairment.

**Conclusions:**

The exact pathophysiology of foreign language syndrome remains unclear, most specifically whether it is a syndrome of its own or a phenotype of emergence delirium. There is still much to be learned, and further research is needed.

## Background

Language switch after an operation or exposure to anesthetic is an exceptional presentation, and there is ambiguity surrounding the presentation and the exact cause. In the literature, some articles associate the language switch with “foreign language syndrome” (FLS), also known as “transient post-anesthetic foreign language fixation” and formerly known as “non-native language syndrome” In FLS, patients switch from their native language and fixate on a second language for a period of time.

To date, there has been no broader analysis of possible mental disturbances with which FLS could be associated. With this paper, we report a case of postoperative language switch (FLS) in a 17-year-old. In addition, we conducted a literature search to find similar cases of language switch. We use this opportunity to share our hypothesis of the clinical position of FLS within the concept of emergence delirium (ED), citing the close similarities in their respective onsets (for example, postoperatively, with possible association to anesthesia), presentations (for example, confusion state), and prognosis (recovery within hours).

## Case presentation

A 17-year-old Dutch Caucasian male was admitted to the hospital following knee surgery for chondral defect of the lateral femoral condyle, after an incident during a game of football. He had no psychiatric or somatic comorbidities, other than factor V Leiden (which was under control), and this was his first time undergoing surgery and anesthesia. His past medical history included greenstick fracture of the distal radius in 2018, a fracture of the basis of the proximal phalanx fifth digit of the right hand in 2014, pneumonia in 2014, and fluctuating idiopathic dysphagia (since grade 3). The patient had no psychiatric history and no relevant medical family history. There was no notable family health history apart from depression on the mother’s side of the family (great grandmother and grandfather). However, he had a psychiatric family history (great grandmother and a grandfather from his mother’s side known with depression). At that time, he was still living at home with his biological parents and he had an older sister (19 years old). He had no relationship. Regarding his education, he was in his final year of high school, with a profile of nature and health.

The patient’s native language was Dutch, and his second language was English. The latter was acquired in a nonbilingual school during regular English classes. He had only spoken English during these classes. He had spoken Dutch exclusively throughout his life, including on the morning of his surgery, and he spoke with a particular southern Dutch dialect (Limburgish). He had no relatives in any English-speaking country, and he had not recently visited any such country. Anesthesia was induced with various medications (Table 1). Upon emergence, the patient was confused and was taken to the recovery room, where a nurse noticed that he was speaking English. He said repeatedly that he was in the United States of America (specifically Utah), despite never having been to the USA. He did not recognize his parents and could neither speak nor understand Dutch. At that time, the nurse had no concerns and thought that the patient was unable to speak Dutch owing to a possible emergence delirium (ED) he was experiencing after the operation. However, a few hours after the surgery, all efforts to elicit a single Dutch word from him had been unsuccessful and psychiatric consultation was sought.

**Table 1:** Summary of cases of foreign language syndrome

Author	Demographics	Type of surgery	Administered medications	Native language (L1)	Second language (L2)	Outcome	Notes	Author theory/conclusion
Webster [5]	55-year-old Caucasian male (New Zealand)	Pharyngoscopy, inversion of the pouch, and a cricopharyngeal myotomy	Premedication: - Midazolam 2 mg Anesthesia - Propofol 3 mg/kg - Rocuronium 1.2 mg/kg Analgesia: - Intermittent boluses of fentanyl (up to a total dose of 1.5 μg/kg) Prophylactic: - Augmentin 1.2 g	English	- Spanish (working knowledge) - Started learning at the age of 37 years - Spoken yearly due to his visits to Chile	- Initially Spanish speaking; understood English and Spanish - Recovered English-speaking ability in 1 hour after sleeping	- Recalled the event and the frustration of not being able to think of an English reply - Did not remember any specific details of what he said in Spanish	- Native and non-native languages are stored in spatially separate areas in the brain - Anesthetics affect the areas differentially, so one language faculty remains active while the other is inactive - Hypoglycemia could be associated - In this case, unclear whether the patient was hypoglycemic at the time of his transient fixation episode
Pollard [6]	64-year-old Caucasian male (from USA)	Bladder cancer presented for radical cystectomy	- Opioid for pain control. Anesthesia: - Propofol (maintained with volatile agents and fentanyl)	American English	Norwegian	- Norwegian Speaking only postoperative - Recovered English-speaking in 5 hours		- Assumption that the event transiently affected L1 area of the brain (English) and spared L2 region (Norwegian) - Only males; possible explanation is lateralization of languages in males (left-dominant activation)
Ward [18]	54-year-old Caucasian male (England)	Arthroscopic surgery for medial meniscectomy	Premedication: - Ranitidine 150 mg - Metoclopramide 10 mg Anesthesia: - Midazolam 2 mg - Propofol 180 mg - Fentanyl 75 mg Other: - Diclofenac suppository 100 mg (inserted rectally)	English	Spanish	- Initially Spanish speaking - Recovered English-speaking once glucose replenished	- No recall of speaking Spanish - Second time speaking Spanish after surgery with general anesthesia - Denied being able to speak it any longer	Suppression of a mother tongue leading to the release of acquired language Possible explanations: *- Hypoglycemia*: a temporal lobe seizure was induced by hypoglycemia and postictal period speech was depressed, allowing the learned speech to emerge *- Anesthesia*, resulting in significant cerebral insult *- Parapsychology*
Cosgrove [19]	Male in his 70s, undocumented race	An open reduction and internal fixation of a fractured tibia	- Fentanyl - Propofol	English	Hindi (learned some phrases in army during World War II)	During the induction, when counting aloud to 30, he began in English and then continued in Hindi	Denied being able to remember or speak Hindi	Language switching due to general anesthesia
Akpek [20]	68-year-old Caucasian male (Czechoslovakian, living abroad)	Unknown		Czechoslovakian	English	- Did not understand English commands - Recovery time not documented		- Main language is primarily stored in “implicit memory systems” of the subcortical regions - Acquired languages stored more diffusely in the cerebral cortex - The role of anesthesia is not yet understood
	Male of undocumented race and age (from Turkey, lived in USA)	Unknown		Turkish	English	- English-speaking only postoperative - Recovered speaking Turkish within 24–28 hours		
Yulia Ivashkov [21]	52-year-old Caucasian male	Elective ankle osteotomy for a malunited tibial fracture		English	French (learned some from his mother who was a native French speaker)	French speaking postoperatively, recovered English-speaking in 1 hour	- Did not recall speaking French during his recovery from anesthesia - Remembered his frustration when everybody was “speaking Russian” and not being able to understand - This was possibly a mistake due to hearing an anesthetist speaking English with a Russian accent	Speech suppression could either (a) be produced by anesthetic agents or (b) be a consequence of other cerebral events of an ischemic nature (embolic or otherwise)
	28-year-old Caucasian male	Right orbital floor blowout fracture was undergoing a fracture repair	- Midazolam - Fentanyl - Propofol - Rocuronium - Maintained with sevoflurane	English	Spanish (studied in primary school but had never used it)	- Understood English and could follow commands but responded in Spanish - Recovered in 25 minutes postoperation	- Had had several similar episodes of conversion to Spanish in the past during occasions of severe alcohol intoxication - The intoxication events had required an emergency medical response, and the medical personnel had noted that he had spoken fluent Spanish during these episodes	
Our case	17-year-old Caucasian male (the Netherlands)	Cartilage repair lateral femoral condyle right	Anesthesia during surgery: - Sufentanil 35 mcg - Morphine 4 mg - Propofol 290 mg + propofol 928 mg 10 mg/ml - Cefazoline 1000 mg - Efedrine 7.5 mg - Paracetamol 1000 mg - Dexamethasone 4 mg - Ondansetron 4 mg - Tranexamic acid 375 mg - Ringer’s lactate 1000 ml	Dutch, southern dialect (Limburgish)	English (acquired in a nonbilingual school during regular English classes)	- Initially English-speaking and did not understand Dutch postoperatively - Recovered English-speaking after 24 hours	In the beginning, was confused and was unaware he was speaking a non-native language	FLS as phenotype of ED

During the mental status examination, which took place approximately 18 hours after surgery, we found a relaxed, 17-year-old, well-groomed boy lying on the bed. We shook hands upon greeting. He made adequate eye contact and was open to communication. His attention could be attracted, and it was maintained well. During the interview, he was able to answer questions, but only in English, spoken with a Dutch accent. He gave only short answers in Dutch and did so with difficulty. His concentration seemed undisturbed. No thought delusions or hallucinations were observed during the conversation. His intelligence was estimated to be average. His mood was cheerful, with a normal affect. His use of the English language seemed adequate; his pronunciation and articulation were clear, with good intonation. His thinking, in terms of form and content, seemed undisturbed. He had a normal facial expression.

Approximately 18 hours postoperatively, the patient was able to understand Dutch but still could not speak it. The physicians involved observed that he spoke English adequately but appeared not to be fluent, while the nurses and his mother observed that he had the ability to speak the language fluently. Approximately 24 hours postoperatively, when some of his friends came to visit, he was able to spontaneously understand and speak Dutch again. Interestingly, during the mental status examination the next day, the boy revealed that he was aware he had been speaking and only able to understand English in the immediate postoperative period. In addition, he remembered that he had been unable to recognize his parents and that he had believed he was in the USA. The neurologist reported no abnormalities in the complete neurological examination. The neurologist saw no indication for further diagnostics; therefore, no electroencephalogram (EEG), neuroimaging, or neuropsychological examinations were performed near the event, and the patient was discharged a day later. Three weeks after discharge from the hospital in a follow-up appointment at the psychiatric outpatient clinic, he reported that he was experiencing no difficulties using the Dutch language. Furthermore, he experienced no other neurological complaints (for example, complaints regarding his senses). There were no changes in his mood and no presentation of anxiety, and his sleep was intact. He did report a decrease in his concentration (including difficulties storing information) or fatigue, especially after the operation. At three follow-up appointments (2 months, 5 months, and 10 months after discharge), these symptoms were gradually improving and there were no new symptoms reported nor observed.

However, almost a year after surgery (when the patient was already 18 years old), a neuropsychological examination was performed due to subjective memory complaints, with the patient indicating that he could not remember things as well as he had done before the surgery. The results of this investigation showed that the patient had a high-performance motivation. His performance in the test was generally average to very good. The patient was able to remember meaningful material to an excellent level, using the Loci method. His memorization of short series of numbers and words was below average. This showed that the patient benefited from repetition. He performed well in terms of vocabulary and visual spatial awareness. The test did not reveal any indication of a cognitive impairment, and the subjective memory complaints may have been due to the patient being fixated on regular forgetfulness.

## Discussion and conclusion

We conducted a literature search in an online electronic database (including PubMed and the reference lists of the found articles), using relevant terms (“foreign language syndrome,” “FLS,” “non-native language,” “rare diseases,” “postoperative anaesthesia,” “general/adverse effects,” “knee surgery,” “drug effects,” “factor V Leiden,” “language physiology,” “Wernicke area/physiology,” “Wernicke area/drug effects,” “monolingualism,” and “multilingualism”) and found eight relevant published cases of FLS. Table 1 summarizes these cases. Child cases of FLS are relatively rare. To the best of our knowledge, most of the reported child cases were presented in news outlets, rather than in scientific journals. Therefore, our case might be the first scientifically reported case of FLS in children/adolescents. Age was also a major difference between the found cases and our own, as all the former were adults—and six of the eight were above the age of 50 years (the age of one case was unknown, and another was 28 years old). Almost all cases—including our own—were male and Caucasian (in two cases, the race was unknown). In six of the cases, the native language was English. In five (including our own), the second language was reported to have been learned as a foreign language, as opposed to the patient being bilingual; the other four cases did not include this information. In five cases (including our own), the episodes occurred after an orthopedic surgery; and in all eight, they lasted between 25 minutes and 28 hours each. Four cases did not recall speaking the other language. Fentanyl, midazolam, and propofol were the most commonly reported anesthetics. No associations indicated whether FLS could be a phenotype of any other postoperative disorder or mental disturbance, such as emergence delirium (ED). As conversion disorder was included in our differential diagnosis (other differentials were ED and FLS), we conducted a brief general search and found three reports of foreign accent syndrome (FAS) being linked to conversion disorder—one of which was written in Japanese, though the abstract was in English [1–3]. FLS should not be confused with FAS, which is “a rare speech disorder consisting of speech rhythm changes perceived by listeners as a foreign accent, and different accents have been reported” [4 p. 1123]. FLS may be similar in presentation, but the onset and clinical course of our clinical case dispute this. In our case, there was no speech production problem, but rather a complete switch of a spoken language and an inability to speak one’s own language.

Although we found only eight relevant cases with a variety of hypotheses and assumptions, it should be noted that the use of non-native languages might go unrecognized by clinical staff who are not familiar with them. The question also arises as to whether our patient was able to speak English fluently. In one case presented by Webster *et al.* the nurse (a Spanish-speaking nurse) reported that the patient’s speech was fragmented and he was repeating a specific single phrase several times [5]. The patient discussed by Pollard *et al.* was not able to speak Norwegian fluently, according to his spouse [6]. Although this was refuted by the anesthesiologist, he was actually from Croatia; thus, Norwegian was not his native language.

Another phenomenon that frequently occurs after operations and anesthetics that could, in our opinion, be related to FLS is ED. We hypothesize that FLS could be a phenotype of ED, rather than a problem of its own. Sikich *et al.* define ED as “a disturbance in a child’s awareness or attention to his/her environment with disorientation and perceptual alterations including hypersensitivity to stimuli and hyperactive motor behaviour in the immediate post anaesthesia period” [7 p. 1139]. The specific mechanism of ED in children remains unknown. Pain and perioperative anxiety may be contributing factors in causing ED [8]. However, this was not applicable to our case. Therefore, we chose not to focus on this further. The possible involvement of anesthesia is another interesting hypothesis, as the incidence of ED is increasing since the introduction of fast-acting volatile agents (for example, sevoflurane and desflurane) [8]. The effect of anesthesia on cognition has been observed in animal studies. In those studies, apoptotic neurodegeneration and long-term cognitive deficiencies have been reported in immature animals exposed to anesthesia [9]. This might explain the experienced lack of concentration in our case.

Dahmani *et al.* reported different possible mechanisms of ED related to anesthesia [8];

Differential recovery rate of brain functions from anesthesia could be caused by the clearance of volatile agents from the central nervous system. It has been hypothesized that the late emergence of cognitive function is the cause of the confusion state in ED. It has also been demonstrated that propofol could be preventive against ED, compared with sevoflurane and desflurane.

The connectivity of brain areas would be susceptible of change while under anesthesia. It has been hypothesized that volatile (such as sevoflurane) and intravenous agents (such as propofol) have several effects on brain networks and might account for differences in recovery manifestations [8]. Based on the first hypothesis, our patient should have had less chance to develop ED, yet the second hypothesis does not exclude the effect of propofol in causing ED.

Some of the found cases report that their patient lacked self-awareness, denied being able to speak in the non-native language, and was unaware that they had spoken or written in a non-native language. These could be signs of confusion and disorientation, as seen in ED. Our patient was initially confused (unable to recognize his family, for example), disoriented in place, and unaware that he was speaking a non-native language. Later, he indicated that he was able to remember not speaking in his native tongue. However, it might be that he only believed he was able to remember because he had been told by clinical staff, family members, and friends what had happened. Language can be affected in delirious patients. Green *et al.* demonstrated that the production of spontaneous speech, word quantity, speech content, and verbal and written language comprehension are impaired in delirious patients compared with cognitively unimpaired patients [10]. Malarbi *et al.* report that irrelevant language, activity, and vocalization are behaviors associated with ED, demonstrating that anesthesia can result in ED and cause possible disturbance in language [11].

An argument may arise regarding recovery time, as the recovery time of FLS can be up to 28 hours, whereas symptoms of ED are thought to occur within 30 minutes of termination of anesthesia and last for 15–30 minutes. However, this is a very common misunderstanding regarding ED, which has been reported to last up to 2 days. The recovery of our patient is thus within this margin.

The exact pathophysiology of FLS remains unclear. Most importantly, we do not know whether it is a syndrome on its own or a phenotype of another syndrome or disorder (for example, ED). This case report sheds light on the possible occurrence of this phenomenon in children not only in adults, as most of the reported cases on FLS. Furthermore, it raises awareness of the probable relations between FLS and ED, which gives the opportunity for better prevention and intervention. As we have demonstrated, ED arises from a combination of factors in which the strongest evidence has been found for undergoing anesthesia and choice of anesthetic. However, patient- and environment-related factors may also have a contributing role, although this is less strongly proven. Non-drug interventions such as preparation for surgery through a tour of the operating room and parental participation in the perioperative process can also play a role in the prevention of ED [12–17]. In addition to non-drug interventions, drug interventions deserve a role in the prevention of ED.

Clinical research on different phenotypes of delirium is important, especially postoperatively and in the emergency ward, to optimize patient care. Thus, there is still much to be learned, and further research is needed.

## Data Availability

Data sharing is not applicable to this article, as no datasets were generated or analyzed during the current study.

## Abbreviations

ED: Emergence delirium
FLS: Foreign language syndrome
FAS: Foreign accent syndrome
